# The Chick Chorioallantoic Membrane as a Xenograft Model for the Quantitative Analysis of Uveal Melanoma Metastasis in Multiple Organs

**DOI:** 10.3390/cells13141169

**Published:** 2024-07-09

**Authors:** Hongtao Liu, Theodora Tsimpaki, Ralitsa Anastasova, Nikolaos E. Bechrakis, Miltiadis Fiorentzis, Utta Berchner-Pfannschmidt

**Affiliations:** Department of Ophthalmology, University Hospital Essen, University of Duisburg-Essen, Hufelandstr. 55, 45147 Essen, Germany; hongtao.liu@uk-essen.de (H.L.); theodora.tsimpaki@uk-essen.de (T.T.); ralitsa.anastasova@uk-essen.de (R.A.); nikolaos.bechrakis@uk-essen.de (N.E.B.); miltiadis.fiorentzis@uk-essen.de (M.F.)

**Keywords:** uveal melanoma, patient-derived xenograft, chick chorioallantoic membrane, experimental metastasis, spontaneous metastasis, liver metastasis, risk factors

## Abstract

Uveal melanoma (UM) is the most common intraocular tumor in adults, and nearly 50% of patients develop metastatic disease with a high mortality rate. Therefore, the development of relevant preclinical in vivo models that accurately recapitulate the metastatic cascade is crucial. We exploited the chick embryo chorioallantoic membrane (CAM) xenograft model to quantify both experimental and spontaneous metastasis by qPCR analysis. Our study found that the transplanted UM cells spread predominantly and early in the liver, reflecting the primary site of metastasis in patients. Visible signs of pigmented metastasis were observed in the eyes, liver, and distal CAM. Lung metastases occurred rarely and brain metastases progressed more slowly. However, UM cell types of different origins and genetic profiles caused an individual spectrum of organ metastases. Metastasis to multiple organs, including the liver, was often associated with risk factors such as high proliferation rate, hyperpigmentation, and epithelioid cell type. The severity of liver metastasis was related to the hepatic metastatic origin and chromosome 8 abnormalities rather than monosomy 3 and BAP1 deficiency. The presented CAM xenograft model may prove useful to study the metastatic potential of patients or to test individualized therapeutic options for metastasis in different organs.

## 1. Introduction

Melanoma originates from transformed melanocytes and can affect various tissues and organs in the body [1]. When it occurs in the uveal tract of the eyes (including the iris 4%, ciliary body 6%, and choroid 90%), it is referred to as uveal melanoma (UM). UM is the most common primary malignant intraocular tumor in adults, with an incidence ranging from approximately two to nine per million, and is associated with ethnicity, age, and latitude [2,3,4]. UM tumors can be treated successfully using several options, including brachytherapy, proton therapy, surgery, or enucleation. Despite reaching a tumor control rate of 90% of UM patients, irrespective of therapy type, half of these patients eventually develop clinical metastasis during the 35 years after primary diagnosis, with a mortality rate of about 40% within 10 years after diagnosis [5,6,7,8]. Therefore, detection and control of UM metastasis are currently at the forefront of research [8].

UM mainly spreads hematogenously, often occurring asymptomatically in the early stages, although a minority can expand via scleral infiltration [9,10]. Based on anatomical structures and blood circulation pathways, UM should primarily metastasize to the lungs. However, in reality, liver metastases are often the first to appear. The majority of metastases occur in the liver (approximately 85%), followed by the lungs (29%), bones (16%), skin (12%), lymph nodes (11%) brain (5%), and other organs (13%) [8,11,12,13,14]. In 32% of UM patients, metastases develop at multiple sites, mainly including the liver [14,15,16]. When metastases arise in the liver, most cases exhibit a diffuse infiltrative growth pattern, while the remaining show a nodular growth pattern [8,15,17]. However, the exact mechanisms and metastatic pathways leading to the primary dissemination of UM to the liver remain unclear.

When UM is first diagnosed, micro-metastases are only found in 1–3% of patients, but five years later, after treatment of the primary tumor, half of all metastases are diagnosed [18,19,20,21,22,23,24]. Larger tumor thickness and maximum basal diameter are risk factors predisposing to metastasis [21]. Furthermore, tumor cell characteristics, such as epithelioid cytology, hyperpigmentation, high mitotic activity, and abnormal genetic profile, are established prognostic risk factors for developing metastasis [8,25]. Tumors of spindle, mixed, and epithelioid cells are present in 48%, 44%, and 8% of patients, respectively [26,27]. Both epithelioid and mixed cell types have higher mortality rates. The five-year survival rates of UM patients were 31% with epithelioid cell type and 24% with mixed cell type. In contrast, patients with the spindle cell type of UM have a better prognosis with a five-year survival rate of 70% [25,27,28,29,30,31,32].

Repeated mutational events and chromosomal aberrations lead to the development of UM tumor growth [33,34]. The most common chromosomal aberrations are monosomy 3 and chromosome 8q gain, which were observed in 42% and 53% of patients, respectively, and were simultaneously found in more than 30% of UM cases [34,35]. The presence of these chromosomal aberrations increases the risk of UM metastases and is associated with an earlier onset and a poorer prognosis [33,34,35,36,37,38,39]. However, other chromosomal aberrations observed in UM such as 6q loss, 9p loss, 1q gain, or 6p gain are not associated with the progression of metastasis [40,41,42]. Pathogenesis of UM is defined by mutations in either G protein subunit alpha Q (GNAQ; 56%), G protein subunit alpha 11 (GNA11; 40%), cysteinyl leukotriene receptor 2 (CYSLTR2; 4%), or phospholipase C beta 4 (PLCB4; 2.5%) [6,43]. The oncogenic proteins lead to an over-activation of G-protein-coupled receptor signaling in the MAPK/ERK pathway and YAP signaling, which is involved in tumor growth and proliferation. However, these mutations show no significant differences in terms of overall survival or metastasis outcomes [44,45,46,47]. This is because mutated GNAQ, GNA11, CYSLTR2, and PLCB4 are considered the primary driver mutations in UM, which initiate tumorigenesis but require the action of secondary driver mutations to promote complete malignant transformation [45,48,49]. Mutations in splicing factor 3B subunit 1 (EIF1AX; 21%), splicing factor 3B subunit (SF3B1; 26%), or BRCA1-associated protein 1 (BAP1; 44%) are considered secondary driver mutations and are nearly mutually exclusive [37,43,44,45,46,50,51]. BAP1 mutations are often associated with monosomy 3 and early risk of metastasis, as metastases usually occur within five years of diagnosis [43,52]. However, tumors with EIF1AX mutations almost always exhibit disomy 3 and appear to have the lowest risk of metastasizing [40,53]. Tumors with mutations in SF3B1 mostly involve disomy 3 and metastasis occurs later, ten years after primary diagnosis [43,54,55].

Animal models play a crucial role in the basic research of UM. Compared to conventional rodent models, the chick embryo chorioallantoic membrane (CAM) model represents an easily accessible and cost-effective alternative that has been used to study various cancer-related processes, including tumor growth and development, metastasis, angiogenesis, and cell invasion [56,57,58,59]. The extraembryonic CAM forms around the fifth day of embryonic development and gradually develops into a rich vascular network that creates an ideal environment for tumor growth after transplantation. The immune system of the chick embryo begins to function at the age of 10–15 days. The lack of immune status in the early stages of development makes it a reliable and stable in vivo model for the study of tumor implantation, growth, and metastasis [59]. The CAM assay has already been used as a preclinical model for the investigation of UM [60]. We have established the CAM assay as a UM xenograft model to test novel therapeutic options for UM tumors [61,62]. However, as metastasis is the most common cause of patient mortality, the aim of the recent study was to exploit the CAM assay for the quantification of UM cell metastasis in vivo. We established both an experimental and a spontaneous CAM metastasis model and performed quantitative real-time PCR (qPCR) to quantify human DNA sequences as a surrogate for UM metastases in chick embryo organs. We investigated the frequency and extent of metastasis caused by a panel of five UM cell lines with different origins and genetic backgrounds. Importantly, we included xenografts with monosomy 3 and BAP1 deficiency, as these genetic profiles are prognostic high-risk factors for the development of metastases in patients. For the first time, our study revealed that each cell line induces a specific metastatic pattern with varying degrees of metastasis in the organs tested, with the liver being the most affected, followed by the eyes, brain, and lungs. The distal lower part of the CAM (LCAM), the liver, and the eyes showed visible signs of pigmented metastasis. Risk factors such as abnormal genetic status, hyperpigmentation, epithelioid cell morphology, and a high proliferation rate were associated with metastasis in multiple organs, including the liver. The model created could be suitable for assessing the metastatic potential of tumors in UM patients in order to predict the risk of metastasis and to test individual therapeutic options in future studies.

## 2. Materials and Methods

### 2.1. Characteristics of Uveal Melanoma Cell Lines and Cell Culture

To obtain representative results in this study, a panel of five different UM cell lines was used, consisting of different genetic profiles and cell characteristics such as morphology and doubling time, which may influence the metastatic cascade (Table 1).

UM cell line 92-1 (CVCL_8607), as well as low passages of primary UM cell lines UPMD2 (CVCL_C258) and UPMM3 (CVCL_C295), were kindly provided by Dr. M. Zeschnigk (Institute of Human Genetics, University Hospital Essen, Essen, Germany). The UM cell line Mel270 (CVCL_C302) was kindly provided by Dr. K. Griewank (Department of Dermatology, University Hospital Essen, Germany). UM cell line MM28 (CVCL_4D15), originated from a hepatic metastasis and was obtained from the American Type Culture Collection (ATCC, Manassas, VA, USA). Short tandem repeat profiling was performed for all cell lines, according to published data (Table 1).

Cell lines 92-1 and Mel270 were maintained in RPMI 1640 medium supplemented with L-Glutamine (GIBCO, Fisher Scientific, Thermo Fisher Scientific Inc., Waltham, MA, USA), and UPMD2 and UPMM3 in Hams/F12 medium supplemented with L-Glutamin and 1.176 g/L NaHCO3 (PAN-Biotech GmbH, Aidenbach, Deutschland). For cell line MM28, RPMI 1640 medium was supplemented with 4.5 g/L D-Glucose, 2.383 g/L HEPES buffer, 1.5 g/L sodium bicarbonate, and 110 mg/L sodiumpyruvate (GIBCO, Fisher Scientific, Thermo Fisher Scientific Inc., Waltham, MA, USA). To each medium, 1% penicillin-streptomycin (5000 U/mL) and 10% fetal calf serum was added. In the case of the MM28 cell line, 20% of FCS was added. All cultured cells were kept in a humidified incubator (37 °C, 5% CO_2_) and the medium was refreshed twice a week.

### 2.2. Chick Embryo Chorioallantoic Membrane (CAM) Metastasis Assay

Fertilized white chick eggs (Lohmann Deutschland, Ankum, Germany) were cleaned with 50% ethanol and incubated in a 400-RD incubatoran incubator (Bruja 3000 digital, Siepmann, Herdecke, Germany) at a temperature of 37.7 °C and a humidity of approximately 60% to induce embryogenesis. On experimental day five of embryonic development (ED 5), 4–6 mL albumin was removed from the apical side of the egg to generate an artificial air chamber between the eggshell and the CAM, thereby lowering the level of the CAM. On ED 6, an oval window of approximately 1.5 cm^2^ was cut into the shell, exposing the upper CAM. The window was covered with adhesive tape and the embryo in ovo was returned to the incubator. On ED 7, the chick embryos were subjected to either an experimental or a spontaneous metastasis model.

Experimental Metastasis Model: UM cells were trypsinized at 37 °C for 2 min, resuspended in their respective medium, and counted with a CellDrop (DeNovix, Wilmington, NC, USA). The cell suspension was centrifuged and the acquired cells were suspended in their respective medium without any supplements. For each chick embryo, 50 µL of the sterile cell suspension containing 0.5 × 10^6^ living cells was directly injected into an allantoic vein of the upper CAM, without causing extensive bleeding by using a 30-gauge needle under a stereomicroscope (Leica M80 Wetzlar, Germany). The experimental metastasis protocol was performed in at least two independent experiments with n = 10–12 chick embryos for each cell line. However, around 50% of the chick embryos died before reaching endpoint ED 11 or ED 18, respectively. The control chick embryos remained without injections of UM cells in at least three independent experiments with n = 3–8 embryos.

Spontaneous Metastasis Model: UM cells were trypsinized, resuspended in their respective medium, and counted as described above. The cell suspension was centrifuged to obtain cell pellets of 1 × 10^6^ living cells. The surface of the CAM was gently lacerated with a spoon in the proximity of a blood vessel bifurcation without causing any bleed and a sterile ring was placed as described before [61,71]. The cell pellets were resuspended in 25 µL Matrigel and pipetted into the middle of the ring, which was removed on the next day. In the following days, the cell pellets on the CAM surface developed into vascularized tumor nodules. The spontaneous metastasis protocol was executed in at least two independent experiments with n = 10–12 embryos for each cell line. However, around 30% of the embryos died before reaching ED 18. The control chick embryos remained without any pellet implantation in at least two independent experiments with n = 3–8 embryos.

After grafting the cells, the window in the eggshell was covered again with adhesive tape, and the chick embryos in ovo were incubated for another 11 days until ED 18. In a short-term experiment, the chick embryos were incubated for 4 days until ED 11. On ED 11 or ED 18, the CAM was inspected, and respective tumor nodules on the CAM were imaged and excised. The embryos were decapitated and the organs such as eyes, brain, liver, lungs, and the LCAM were dissected. The organs were stereomicroscopically inspected for signs of tumor growth and brown pigmentation of UM tumor cells. Images were taken using a stereomicroscope Leica M80 equipped with a camera IC80 HD (Leica, Wetzlar, Germany). For quantitative evaluation of metastasis, each individual organ was weighted, frozen on dry ice, and stored at −80 °C until further processing.

### 2.3. Extraction of Genomic DNA

Genomic DNA was extracted either from each individual chick organ or from UM cell lines by using the Purgene DNA purification System (Gentra System, Minneapolis, MN, USA) in accordance with the manufacturer’s instructions. Each organ was thawed on ice, minced, and homoginated by using an IKA T10 basic ULTRA-TURRAX (IKA-Werke, Staufen, Germany). Genomic DNA was extracted from 25 mg of each homogenate by using the Invitrogen PureLink Genomic DNA Mini Kit (Thermo Fisher Scientific, Life Technology Corp. Carlsbad, CA, USA) following the manufacturer’s instructions. Genomic DNA concentration was measured by using a Thermo Scientific NanoDrop 2000 Spectrophotometer (Thermo Fisher Scientific, Waltham, MA, USA).

### 2.4. Real-Time PCR of Genomic DNA

UM cells present in chick tissues were detected by qPCR amplification of human *alu* repeat sequences (huALU) present in the genomic DNA extracted from each chick organ based on the method of Zijlstra et al., 2002 [72]. The huALU were targeted using a specific primer pair with sense 5′ ACG CCT GTA ATC CCA GCA CTT 3′ and antisense 5′ TCG CCC AGG CTG GAG TGC A 3′ [72]. In order to detect chick cells, the chick *GAPDH* sequences (chGAPDH) were targeted using a primer pair with sense 5′ GAG GAA AGG TCG CCT GGT GGA TCG 3′ and antisense 5′ GGT GAG GAC AAG CAG TGA GGA ACG 3′ [72]. Each qPCR reaction was performed in duplicates using 40 ng of genomic DNA, 0.4 µM of each primer, and 1× Takyon No Rox SYBR MasterMix dTTP Blue containing 2.5 mM MgCl_2_ (Eurogentec, Seraing, Belgium). PCR reactions were conducted with StepOnePlus Real-Time-PCR System (Applied Biosystems, Waltham, MA, USA) under the following conditions: polymerase activation at 95 °C for 2 min followed by 30 cycles of denaturation at 95 °C for 30 s, annealing at 63 °C for 30 s, and extension at 72 °C for 30 s. Each qPCR assay included a non-template control, genomic DNA of organs taken from control chick embryos as UM cell negative controls, genomic DNA extracted from UM cell lines as positive controls, and genomic DNA of organs taken from chick embryos, which underwent metastasis protocols.

The resulting qPCR products were analyzed with the 2^−ΔΔCT^ method by using the formula ΔΔCt = metastatic embryo organ (Ct huALU − Ct chGAPDH)—control chick embryo organ (Ct huALU − Ct chGAPDH). The resulting values reflect the relative fold of huALU sequences in a metastatic organ over a control organ and serve as a surrogate for the relative amount of metastasis. Samples with values <1 were below chick embryo control values and considered negative for metastases. The number of organs positive for metastasis is given in percent (%). In addition, the percentages of chick embryos that tested positive for metastasis for one or multiple embryonal organs were analyzed separately.

### 2.5. Statistical Analysis

Statistical analysis of the data was performed using Graph Pad Prism 8.4.3 software (GraphPad Software Inc., San Diego, CA, USA). An unpaired *t*-test was applied when comparing the two groups. One-way Anova was used when multiple groups were compared. When data did not meet the assumptions of normality, the Mann–Whitney test was used to compare two groups and multiple groups were compared with Kruskal–Wallis with Dunn’s multiple comparison test. Significance levels are indicated as follows: * *p* < 0.05, ** *p* < 0.01, *** *p* < 0.001; **** *p* < 0.0001, and *p* values *p* > 0.05 are not significant and not indicated.

## 3. Results

### 3.1. Experimental Metastasis of UM Cell Types in the CAM Model

**Figure 1:**
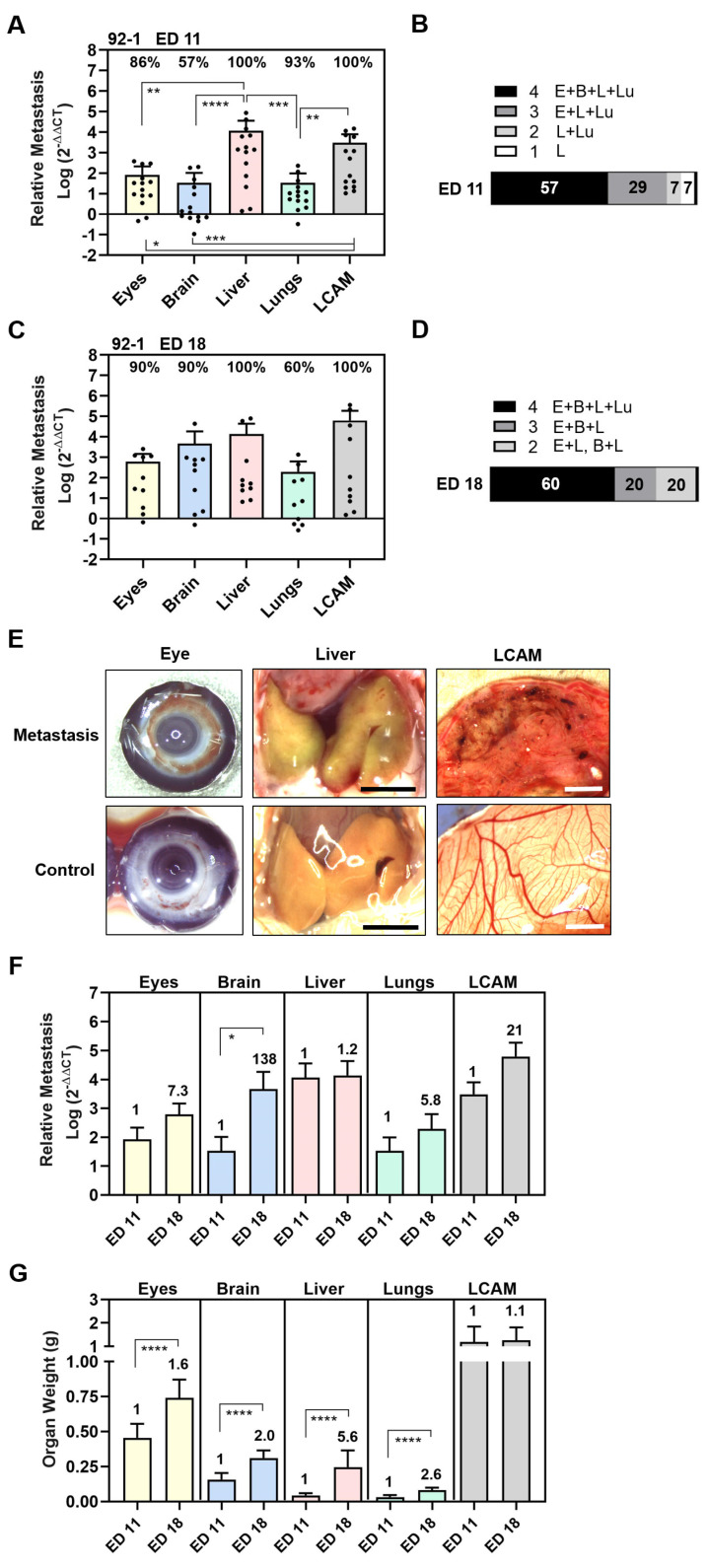
Experimental metastasis of UM cell line 92-1 in the CAM assay. Living cells were injected into the chick circulation and the induced colonization of organs was evaluated by means of qPCR at experimental day (**A**,**B**) ED 11 or (**C**,**D**) ED 18. (**A**,**C**) The relative amount of metastasis of each organ was determined. Mean data (±SD) are shown for ED 11 (n = 14) and ED 18 (n = 10). Percentages of organs that tested metastasis-positive are indicated. Statistical analysis was performed using a Kruskal–Wallis multi-comparison test, * *p* < 0.05, ** *p* < 0.01, *** *p* < 0.001, **** *p* < 0.0001. (**B**,**D**) Percentages of individual chick embryos that tested metastasis-positive for the indicated number of organs were determined (1 to 4 embryonal organs: E = Eyes, B = Brain, L = Liver, Lu = Lungs). (**E**) Representative images of eyes, livers, and LCAMs obtained from either metastatic or control chick at ED 18 (scale bar represents 5 mm). Visible signs of metastasis were brown iris hyperpigmentation in the eyes, green-brownish colored liver areas, and dark-brown metastatic foci in the mesenchyme of LCAM. However, only the LCAM presented visible signs of metastasis at ED 11. (**F**) Comparison of amounts of metastasis at ED 11 and ED 18. Fold induction at ED 18 is indicated. Statistical analysis was performed using a Kruskal–Wallis test, * *p* < 0.05. (**G**) Mean weights of organs (±SEM) obtained at ED 11 (n = 14) or at ED 18 (n = 10) are shown. The fold induction at ED 18 is indicated. Statistical analysis was performed using an unpaired *t*-test, **** *p* < 0.0001.

To establish the experimental metastasis assay, we started with a 92-1 cell line, known to be one of the most frequently used UM cell lines in animal models [73]. To induce experimental metastasis, we injected 0.5 × 10^6^ cells into a vein of the chick allantois on ED 7. Subsequently, chick embryos were either incubated for 4 days until ED 11 or were incubated for 11 days until ED 18. Analysis of qPCR revealed that human sequences, and thus 92-1 cells, could be detected in all tested organs already after 4 days of incubation at ED 11 (Figure 1).

We found that 86% of eyes, 100% of livers, 93% of lungs, and 100% of LCAMs were positive for 92-1 cells, while only 57% of brains were 92-1-positive at ED 11 (Figure 1A). The relative amount of metastases was statistically significantly highest in the livers and LCAMs, followed by the eyes and lungs. The lowest amount of metastases was detectable in the brains (Figure 1A). The amount of liver metastasis was 138 times higher than orthotropic growth in the eyes and 342 times higher than metastases in the lungs or brains (mean values: livers 11,541, eyes 83, lungs 34, and brains 34), indicating that 92-1 cells caused predominantly liver metastases at ED 11 (Figure 1A). As a result, 57% of the chick embryos were 92-1-positive for all tested organs, 37% were positive for three organs (eyes, liver, and lungs), 7% for two organs (liver and lungs), while one animal was positive only for the liver (Figure 1B).

After a prolonged incubation period until ED 18, the 92-1 metastasis persisted in 100% of the livers and 100% of the LCAMs (Figure 1C). Stereomicroscopically, dark brown metastatic foci (diameter < 1 mm) caused by the pigmented 92-1 cells were detectable in the mesenchymal tissue of 50% of the LCAMs, indicating extravasation and proliferation of tumor cells, while all control LCAMs were completely free of any visible metastatic foci (Figure 1E). In comparison, only 43% of the LCAMs showed metastatic foci at early time point ED 11. Furthermore, at ED 18, at least 10% of the livers had macroscopically dark green-brown pigmented areas, indicating the presence of metastatic lesions in the liver tissue, compared to the homogeneously yellow-stained control livers at ED 18 (Figure 1E). Further, qPCR analysis revealed that orthotropic growth of 92-1 cells persisted in 90% of the eyes. We observed brown hyperpigmentation of the iris in 10% of chick eyes, suggesting visible signs of iris melanoma (Figure 1E). Furthermore, qPCR analysis revealed that only 60% of lungs remained positive for 92-1 cells, while metastasis in the brains increased dramatically in up to 90% of embryos until ED 18 (Figure 1C). None of these organs presented visible signs of metastasis when observed stereomicroscopically, most likely indicating that 92-1 metastasis caused extended tissue abnormalities predominantly in LCAMs, liver, and eyes. Indeed, the mean amount of metastases was higher in the livers compared to all other organs while the lungs were the least affected, although not statistically significantly different due to the high variability of metastasis between the embryos (Figure 1C). The mean number of liver metastasis was 3-fold higher than brain metastasis, 23-fold higher than orthotropic growth in the eyes, and 71-fold higher than metastasis in the lungs (mean values: liver: 13,804, brain: 4640, eyes: 607, and lungs: 196). Overall, at ED 18, we found that 60% of the chick embryos were metastasis-positive in all embryonic organs, while 20% of the chick embryos developed metastases in two (10% eyes and liver, 10% brain and liver) or three organs (eyes, brain, and liver) (Figure 1D).

Moreover, a direct comparison between the development time points revealed that the mean amount of metastases in organs increased until ED 18 (Figure 1F). The prolonged incubation time led to a 7.3-fold increase in orthotropic growth in the eyes, a 1.2-fold increase in liver metastasis, a 5.8-fold increase in lung metastasis, and a 21-fold in LCAM metastasis (Figure 1F). Of note, the mean amount of brain metastasis increased statistically significantly up to 138-fold, most likely indicating that brain metastasis progressed at a later time point during the experimental course (Figure 1F). In addition, the embryo was developing, so that the growth of the organs progressed simultaneously (Figure 1G). The mean weight of the embryonic organs increased statistically to different extents: the mean weight of the eyes increased 1.6-fold, that of the brains 2.0-fold, that of the livers 5.6-fold, and that of the lungs 2.6-fold. The LCAM weight increased only slightly by 1.1-fold. Although the mean weight of the livers increased 5.6-fold, the amount of liver metastases increased only slightly to 1.2-fold, possibly due to the faster growth of normal liver tissue than liver metastases.

**Figure 2:**
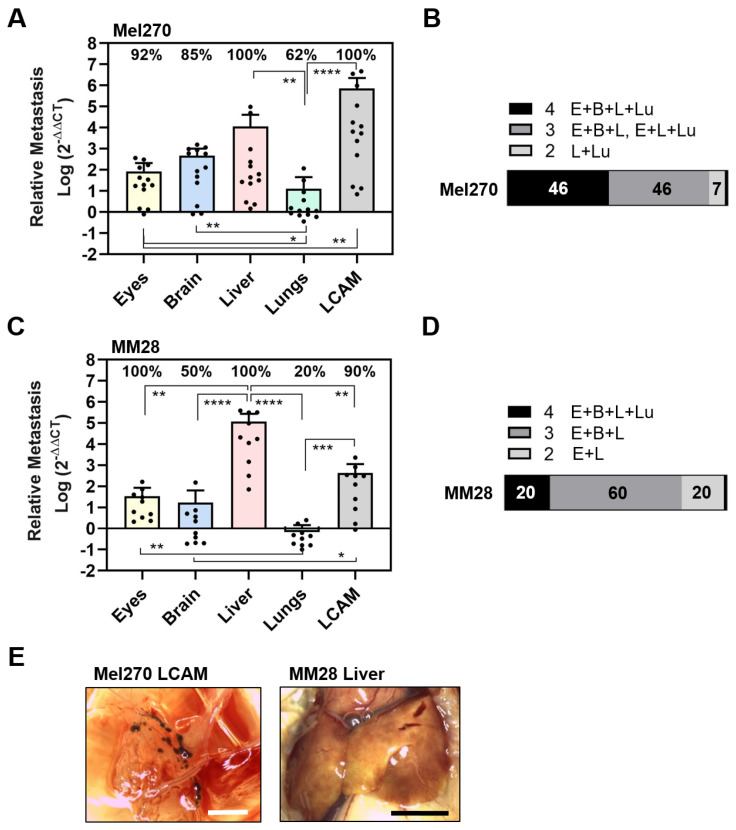
Experimental metastasis of cell lines Mel270 and MM28 in the CAM assay. Living cells of each cell line were injected into the circulation of chick embryos and resulting metastasis was evaluated at ED 18. (**A**–**D**) The relative amount of metastasis was determined by qPCR for cell line (**A**,**B**) Mel270 or (**C**,**D**) MM28. (**A**,**C**) Mean data of amounts of metastasis (±SD) are presented for Mel270 (n = 13) or for MM28 (n = 10). Percentages of organs that tested positive for metastasis are indicated. Statistical analysis was performed using a Kruskal–Wallis multiple comparison test, * *p* < 0.05, ** *p* < 0.01, *** *p* < 0.001, **** *p* < 0.0001. (**B**,**D**) Percentages of individual chick embryos that tested metastasis-positive for the indicated number of organs were determined (1 to 4 organs: E = Eyes, B = Brain, L = Liver, Lu = Lungs). (**E**) Representative images of the LCAM or the liver obtained from a Mel270 or MM28 metastatic chick embryo are shown (scale bar represents 5 mm). The LCAM revealed dark brown metastatic foci alongside vessels in the mesenchyme, while the livers presented with brown-colored areas, suggesting parts of the livers were metastatic.

Since cell line 92-1 originates from a large, untreated primary tumor, we wanted to investigate the metastatic potential of two other cell lines with different origins. Mel270 was derived from a recurrent primary tumor after radiotherapy, while MM28 originated from a liver metastasis (Table 1). We chose the longer incubation period of 11 days to investigate persistent metastasis in ED 18 (Figure 2).

The Mel270 cell line caused an almost similar pattern of metastasis in the embryonic organs as the 92-1 cell line, affecting 100% of the livers, 100% of the LCAMs, 92% of the eyes, 85% of the brains, and 62% of the lungs (Figure 2A). Similarly, the extent of metastasis was highest in the livers and the LCAM. Metastasis in the livers was 133 times higher than orthotropic growth in the eyes (mean values: liver 11,422, eyes 86). However, the amount of metastasis in the lungs was significantly lower compared to all other organs (Figure 2A). In addition, the extent of metastasis was higher in the LCAMs compared to all other organs and differed significantly from orthotropic growth in the eyes. However, Mel270 cells formed dark brown metastatic foci in LCAM tissue in only 15% of the chick embryos. Here, we observed metastatic foci located along the vessels (Figure 2E). Overall, the qPCR analysis revealed that 46% of the chick embryos developed metastases in all organs, 46% of the chick embryos were metastasis-positive in three organs, and 7% were positive in two organs, liver and lung.

Next, we analyzed the metastatic activity of the MM28 cell line in the experimental CAM model (Figure 2C,D). We found that MM28 cells caused a different spectrum of organ metastases compared to 92-1 or Mel270 cells. Interestingly, 100% of the eyes and 100% of the livers were positive, while only 90% of the LCAMs, 50% of the brains, and 20% of the lungs were positive for metastases (Figure 2C). In addition, compared to 92-1 and Mel270, the extent of induced metastasis by MM28 was lower in all organs except the liver (Figure 2C). The number of metastases was 3432-fold higher in the livers than in the eyes (mean values: liver 117,134, eyes 34). In addition, compared to 92-1 or Mel270, the MM28 cells caused an 8.5 or 10-fold higher amount of metastasis in the livers (mean values of livers: MM28: 117,134, 92-1: 13,804, Mel270: 11,422). Stereomicroscopically, we observed dark brown-pigmented metastatic areas in 10% of the livers (Figure 2E). In addition, brown-pigmented metastatic foci were observed in 20% of the LCAMs. The qPCR analysis revealed that only 20% of the chick embryos developed MM28 metastases in all organs tested, 60% in three organs, and 20% in two organs (eyes and livers) (Figure 2D). The data indicated that MM28 cells more frequently caused ocular metastases and higher secondary growth in the liver compared to cell lines of primary origin.

**Figure 3:**
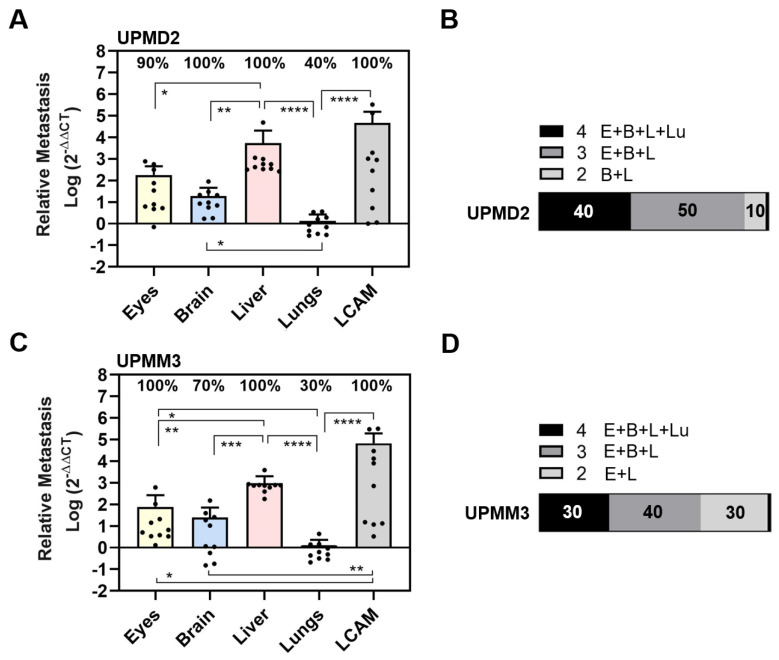
Experimental metastasis of primary cell lines UPMD2 and UPMM3 in the CAM assay. Living cells of each cell line were injected into the circulation of chick embryos. The relative amount of metastasis was determined by qPCR at ED 18. (**A**,**B**) Relative amount of metastasis caused by UPMD2. (**C**,**D**) Relative amount of metastasis caused by UPMM3. Mean data (±SD) are presented for UPMD2 (n = 10) or for UPMM3 (n = 10). Percentages of organs that tested positive for metastasis are indicated. Statistical analysis was performed using a Kruskal–Wallis multiple comparison test, * *p* < 0.05, ** *p* < 0.01, *** *p* < 0.001; **** *p* < 0.0001. (**B**,**D**) Percentages of individual chick embryos that tested metastasis-positive for the indicated number of organs were determined (1 to 4 organs: E = Eyes, B = Brain, L = Liver, Lu = Lungs).

Since MM28 had a different metastatic spectrum, we hypothesized whether the observed differences were due to the hepatic metastatic origin of MM28 or whether the cytogenetic status of chromosome 3 aberration/BAP1 deficiency played a role (Table 1). Therefore, we next investigated the experimental metastasis of two primary UM cell lines exhibiting either genetic disomy 3/BAP1 positivity (UPMD2) or monosomy 3/BAP1 deficiency (UPMM3). Both cell lines presented a relatively similar pattern of organ metastasis characterized by predominant metastasis of the liver, although lower amounts were detected compared to the other tested cell lines, 92-1, Mel270, or MM28 (Figure 3).

Specifically, the UPMD2 cell line caused metastases in 100% of the LCAMs, 100% of the livers, 100% of the brains, 90% of the eyes, and 40% of the lungs (Figure 3A). We found larger amounts of metastases in the LCAMs and livers, while eyes and lungs were less affected by UPMD2 cells. Liver metastasis was 30 times higher than orthotropic growth in the eyes (mean values: liver 5360, eyes 180) (Figure 3A). Chick embryos injected with UPMD2 also showed visible signs of metastasis in 20% of the LCAMs and 10% of each of the livers and eyes. Altogether, the qPCR analysis showed that 40% of the chick embryos developed metastases in all organs, 50% in three organs, and 10% in two organs (brains and livers) (Figure 3B).

The UPMM3 cell line caused metastases in 100% of LCAMs, 100% of livers, 100% of eyes, 70% of brains, and 30% of lungs (Figure 3C). Liver metastasis by UPMM3 was statistically significant and up to 9.5 times higher than orthotropic growth in the eyes (mean values liver: 740, eyes: 78). However, the amount of metastasis in eye or livers metastases was lower with UPMM3 than with UPMD2. Furthermore, the UPMM3-injected chick embryos presented no visible signs of organ metastasis. The qPCR analysis of metastasis in each individual chick embryo revealed that 30% of embryos developed metastasis in all organs and 40% of embryos developed metastasis in three organs. Interestingly, 30% of the chick embryos developed metastasis in only two organs, the eyes and liver (Figure 3D).

Apparently, the monosomy 3/BAP1-deficient cell line UPMM3 had a similar or even lower metastatic potential than the disomy 3/BAP1-positive UPMD2 cell line in the experimental CAM model. However, the cell lines UPMM3 and MM28 with chromosome 3 aberrant/BAP1 deficiency showed a higher frequency of metastases in the eyes (100%) and a higher percentage of chick embryos with metastases only in the eyes and liver (UPMM3: 30%, MM28: 20%, compared to 92-1: 10%, all others 0%).

### 3.2. Spontaneous Metastasis of UM Cell Types in the CAM Model

**Figure 4:**
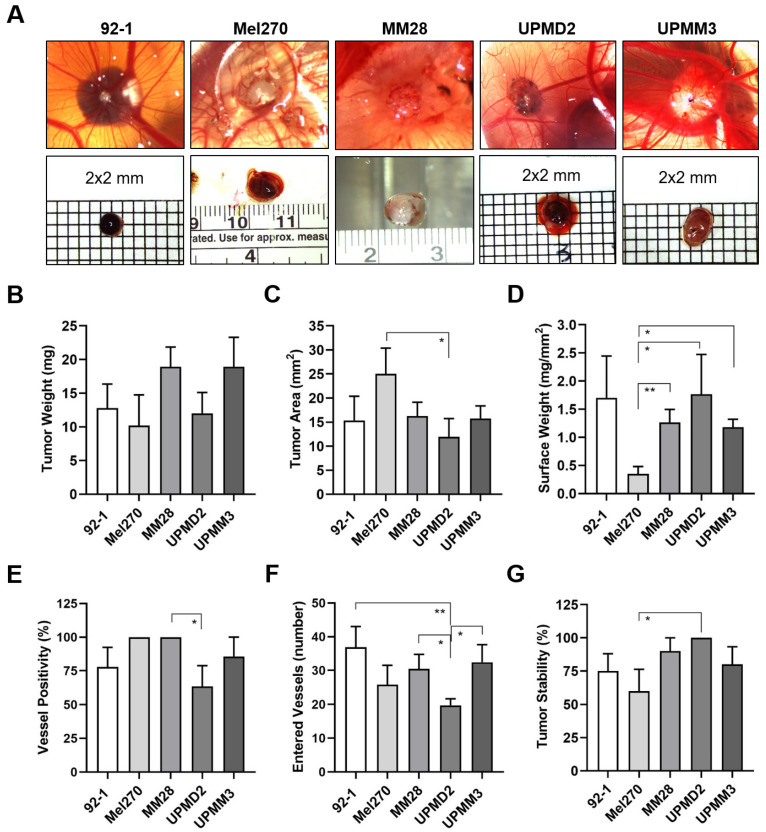
Various UM cell lines formed tumors with different appearances on the CAM. Living cells of each cell line 92-1, Mel270, MM28, UPMD2, or UPMM3 were inoculated as a pellet onto the CAM. Appearance and morphology of resulting tumors were analyzed at ED 18. (**A**) Representative stereomicroscopic images of tumors on the CAM (upper panel) and excised tumors (lower panel) are shown, magnification 0.75×. (**B**) The mean weights of excised tumors are given (mg). (**C**) Basal areas of tumors were measured (mm^2^). (**D**) The tumor surface weight was calculated by dividing tumor weight by the basal area (mg/mm^2^). (**E**) Percentages of tumors that were vessel positive by stereomicroscopy (%). (**F**) Embryonic vessels entering a distinct tumor on the CAM (“vascular star”) were counted (number). (**G**) Percentages of form-stable tumors present on the CAM by ED 18 (%). Data are presented as means (±SEM) of cell lines 92-1 (n = 12), Mel270 (n = 10), MM28 (n = 10), UPMD2 (n = 11) or UPMM3 (n = 10). Statistical analysis was performed using a Kruskal–Wallis multiple comparison test, * *p* < 0.05, ** *p* < 0.01.

To explore the invasive capacity and the ability of cell lines to intravastate, we established a spontaneous metastatic CAM model. Therefore, we inoculated 1 × 10^6^ cells onto the CAM to trigger primary tumor formation. At ED 18, all cell lines 92-1, Mel270, MM28, UPMD2, and UPMM3 had formed vascularized tumors on the CAM with different appearances, sizes, and vascularization (Figure 4).

The tumors formed by the epithelioid cell types 92-1 or UPMD2 were mainly black-brown pigmented and dome-shaped nodules, while the spindle-shaped cell line Mel270 formed red-brown pigmented flat tumors (Figure 4A). The mean weights of the latter tumors did not vary significantly and were between 10 and 13 mg (Figure 4B). However, Mel270 tumors had a significantly larger mean basal area (25 mm^2^) than UPMD2 (12 mm^2^) (Figure 4C). In addition, Mel270 tumors had the lowest surface weight, confirming their flat morphology (Figure 4D). The dome-shaped tumors formed by the mixed cell types UPMM3 or MM28 appeared pale-brownish pigmented (UPMM3) or amelanotic (MM28) (Figure 4A). MM28 and UPMM3 tumors tend to reach a higher weight (mean weights 19 mg) compared to the tumors of the other cell lines (10–13 mg), whereas the basal area did not vary compared to the other cell lines (Figure 4B,C). Stereomicroscopically, all of the Mel270 and MM28 tumors were vascularized, while only 64% of the UPMD2 tumors appeared macro-vessel positive (Figure 4E). In addition, we observed that the tumors stimulated changes in the CAM vascularization during the experimental course, as chick embryo blood vessels converged radially towards the tumors to form a so-called vascular star. The mean number of stereomicroscopically visible chick embryo vessels entering a tumor was significantly lower for UPMD2 tumors (20 vessels) compared to the tumors of the other cell lines 92-1, UPMM3, or MM28 (37, 32, or 30 vessels), while the number of vessels entering the Mel270 tumors appeared in-between (26 vessels) (Figure 4F). However, the efficiency of forming distinct tumor nodules that remained stable in one piece until ED 18 (tumor stability) was highest for UPMD2 (100%) and lowest for Mel270 (60%) (Figure 4G). In addition, we noticed that 25% of 92-1 and 17% of UPMM3 tumors grew below the surface of the CAM.

**Figure 5:**
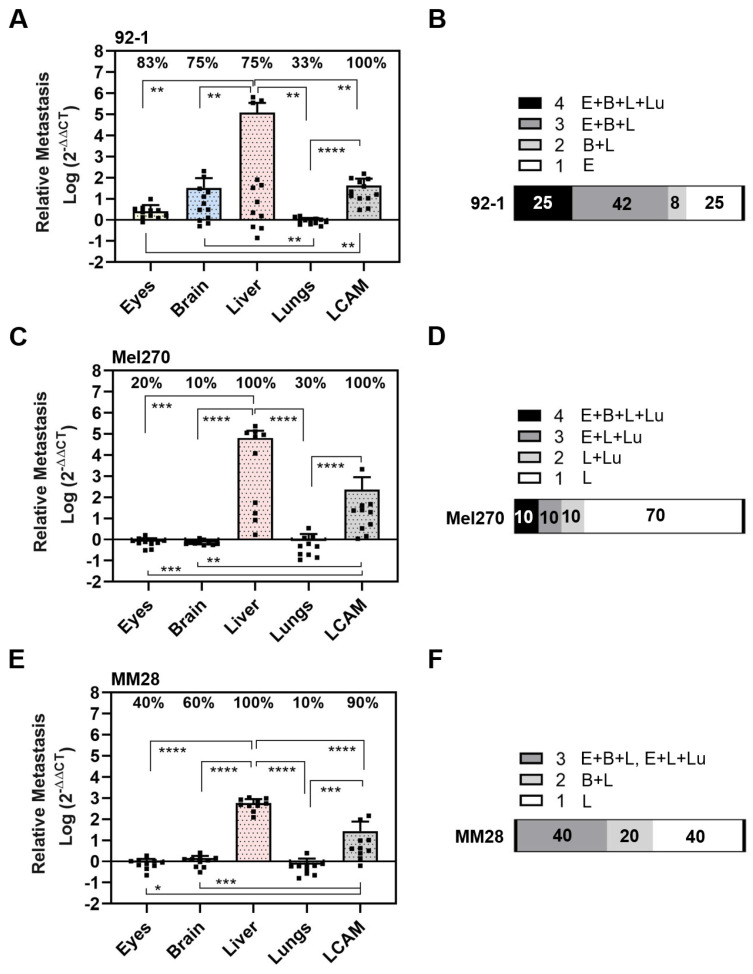
Spontaneous metastasis of UM cell lines 92-1, Mel270, and MM28 in the CAM model. Living cells of each cell line were inoculated on the CAM. At ED 18, the relative amount of metastasis was determined for cell lines (**A**,**B**) 92-1, (**C**,**D**) Mel270, or (**E**,**F**) MM28. Mean data (±SD) are presented for 92-1 (n = 12), Mel270 (n = 10) or MM28 (n = 10). Percentages of organs that tested metastasis-positive are indicated (%). Statistical analysis was performed using a Kruskal–Wallis multiple comparison test, * *p* < 0.05, ** *p* < 0.01, *** *p* < 0.001; **** *p* < 0.0001. (**B**,**D**,**F**) Percentages of individual chick embryos that tested metastasis-positive for the indicated number of organs (1 to 4 organs: E = Eyes, B = Brain, L = Liver, Lu = Lungs).

The tumors of the respective cell lines 92-1, Mel270, or MM28 led to an individual pattern of spontaneous organ metastasis up to ED 18 (Figure 5).

The 92-1 cells frequently disseminated to the LCAMs (100%), eyes (83%), brains (75%), and livers (75%), and least frequently to the lungs (33%). However, compared to all other organs, including the LCAMs, the significantly highest amount of metastasis was found in the livers (Figure 5A). Spontaneous liver metastasis was 46,651 times higher than that of the eyes, 3675 times higher than that of the brains, and 134,770 times higher than that of the lungs (mean amount of metastases: liver: 121,293, eyes: 2.6, brain: 33, and lungs: 0.9). Stereomicroscopically visible signs of metastasis, such as brown pigmentation, were observed in 17% of the LCAMs and in 8% each of the livers and eyes. Altogether, analysis of qPCR revealed that 25% of the chick embryos had metastases in all organs, 42% of the chick embryos had metastases in three organs, 8% of the chick embryos had metastases in the brain and liver, while 25% of the embryos developed orthotropic growth in the eyes only (Figure 5B).

In contrast, Mel270 cells disseminated frequently to LCAMs (100%) and livers (100%), but to a much lesser extent to all other organs such as the eyes (20%), brains (10%), and lungs (30%) (Figure 5C). Of note, the amount of metastasis was much higher in the liver than in the LCAM or any other organ. The spontaneous metastasis of the livers was 81,751 times higher than that of the eyes, 93,430 times higher than that of the brains, and 81,751 times higher than that of the lungs (mean number of liver metastases: 65,401, eyes: 0.8, brains: 0.7, and lungs: 0.8). Remarkably, 70% of the chick embryos showed metastases exclusively in the livers, indicating a higher selectivity for the liver in the spontaneous model (Figure 5D). Visible signs of Mel270 metastasis were observed in 10% of each liver or LCAM.

The MM28 cells metastasized mainly in the livers (100%) and LCAMs (90%), followed by the brains (60%), eyes (40%), and very few lungs (10%) were positive for MM28 cells (Figure 5E). Similar to 92-1 and Mel270, the amount of metastasis was highest in the livers compared to the other organs. Spontaneous liver metastasis was 658 times higher than that of the eyes, 493 times higher than that of the brain, and 846 times higher than that of the lungs (mean amount of metastases: livers: 592, eyes: 0.9, brains: 1.2, and lungs: 0.7). In addition, no chick embryo developed MM28 metastases in all embryonic organs, 40% of the chick embryos had metastases in three organs, and a further 20% of the chick embryos had metastases in two organs (brains and livers) (Figure 5F). Notably, 40% of the chick embryos developed metastasis solely in the livers (Figure 5F). However, the mean number of liver metastases was significantly lower with MM28 compared to 92-1 or Mel270 (0.005 or 0.009 times lower than with 92-1 or Mel270, respectively). Nevertheless, MM28 transplanted chick embryos presented visible signs of metastasis in each 10% of livers or LCAMs.

**Figure 6:**
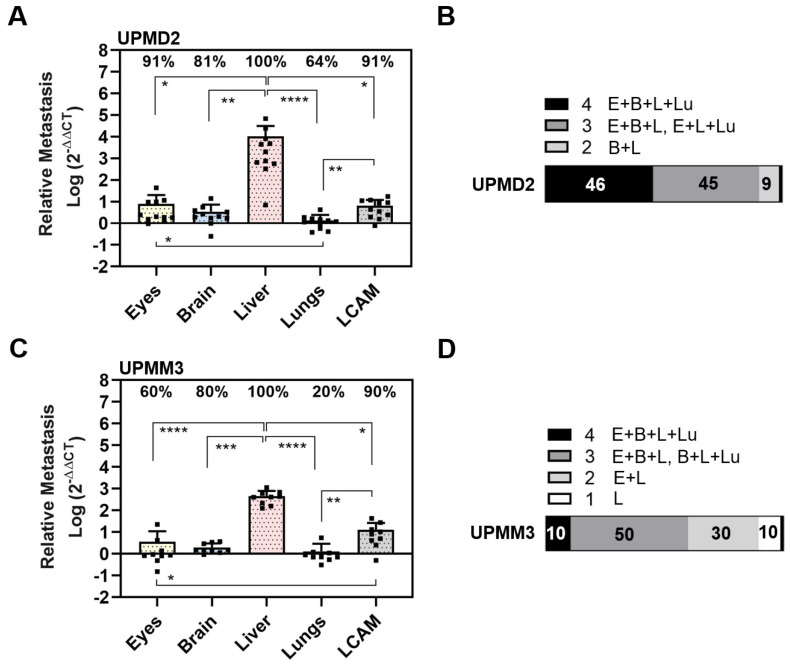
Spontaneous metastasis of primary UM cell lines UPMD2 and UPMM3. Living cells were inoculated on the CAM. At ED 18, the relative amount of metastasis was determined with qPCR for cell line (**A**,**B**) UPMD2 or (**C**,**D**) UPMM3. Mean data (±SD) are presented for UPMD2 (n = 11) or UPMM3 (n = 10). Percentages of organs that tested metastasis-positive are indicated. Statistical analysis was performed using a Kruskal–Wallis multiple comparison test, * *p* < 0.05, ** *p* < 0.01, *** *p* < 0.001, **** *p* < 0.0001. (**B**,**D**) Percentages of individual chick embryos that tested metastasis-positive for the indicated number of organs (1 to 4 embryonal organs: E = Eyes, B = Brain, L = Liver, Lu = Lungs).

We then analyzed the spontaneous metastasis of primary cell lines UPMD2 and UPMM3 (Figure 6).

In detail, UPMD2 cells metastasized in the livers (100%), eyes (91%), and LCAMs (91%), followed by the brains (81%) and lungs (64%) (Figure 6A). Similar to the other cell lines, the amount of metastasis was highest in the liver. Spontaneous liver metastasis was 134,782 times higher than that of the eyes, 3004 times higher than that of the brains, and 7509 times higher than that of the lungs (mean amount of metastases in the liver: 10,513, eyes 7.8, brain 3.5, and lungs 1.4). In addition, orthotropic growth in eyes was most frequent with UPMD2 among all cell lines in the spontaneous setting (Figure 5 and Figure 6). In 9% of the chick embryos, we observed hyperpigmentation of the iris, and in a further 9% of the chick embryos, we found micro-metastatic foci in the LCAM tissue. Overall, the qPCR analysis revealed that 46% of the chick embryos developed metastases in all organs, 45% in three organs, and 9% in two organs (brains and livers) (Figure 6B).

UPMM3 cells metastasized predominantly in the livers (100%) and in the LCAMs (90%), followed by the brains (80%), eyes (60%), and lungs (20%). As well, UPMM3 cells induced the highest amount of metastasis in the livers compared to all embryonal organs. The amount of liver metastasis was 464 times higher than that of the eyes, 129 times higher than that of the brains, and 232 times higher than that of the lungs (mean amount of metastases: liver: 464, eyes: 5.6, brain 2.0, and lungs 1.3). However, compared to all other cell lines the amount of liver metastasis was the lowest. Nevertheless, 10% of the chick embryos presented metastatic pigmentation in the livers or LCAMs. In sum, qPCR analysis revealed that only 10% of the chick embryos developed spontaneous metastasis in all organs, 50% of the chick embryos presented metastasis in three organs, and 30% of chick embryos were metastasis-positive for the two organs, eyes, and livers. Another 10% of the chick embryos developed metastasis solely in the livers (Figure 6D).

In summary, in the spontaneous model, the disomic cell lines 92-1, Mel270, and UPMD2 caused more frequent metastases in multiple organs and developed more liver metastases than the cell lines UPMM3 or MM28 with aberrant chromosome 3 and BAP1 deficiency.

### 3.3. Comparison of UM Metastasis Activity in Experimental and Spontaneous Model

**Figure 7:**
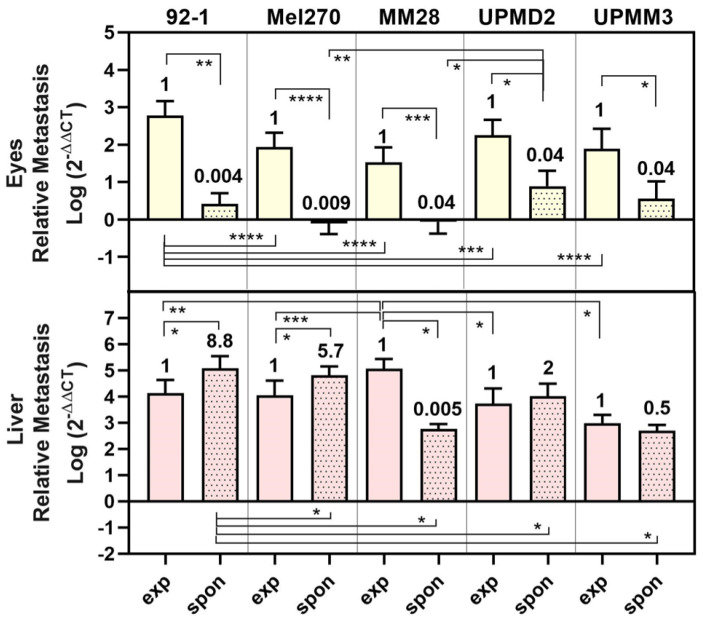
Comparison of metastasis in eyes and livers in experimental and spontaneous model. The mean amount of relative metastasis caused by each cell line 92-1, Mel270, MM28, UPMD2, or UPMM3 in either experimental (exp) or spontaneous (spon) model are presented. Fold change of spontaneous metastasis vs. experimental metastasis is indicated. Statistical analysis was performed using a one-way Anova, Mann–Whitney test, or Kruskal–Wallis test, * *p* < 0.05, ** *p* < 0.01, *** *p* < 0.001, **** *p* < 0.0001.

The experimental and spontaneous models represent two fundamentally different approaches to studying metastasis. However, since we used the same cell line in both models, it seems interesting to compare the outcome of metastasis in the two models for the respective cell line under the given conditions. Overall, the mean amount of metastases was higher in the experimental than in the spontaneous model among eyes, brains, lungs, and LCAMs (Figure 1, Figure 2 and Figure 3 vs. Figure 5 and Figure 6). However, this was not the case for the liver metastasis. We plotted all data obtained for the eyes and liver in the experimental and spontaneous models comparatively in a figure (Figure 7).

The metastasis of eyes was significantly lower (0.04- to 0.009-fold) for all cell types in the spontaneous model than in the experimental model (Figure 7). Similarly, metastasis of livers was significantly lower in the spontaneous model than in the experimental model for cell types MM28 (0.005-fold) or UPMM3 (0.5-fold). In contrast, the disomy 3/BAP1 positive cells 92-1, Mel270, and UPMD2 caused even higher amounts of liver metastasis in the spontaneous model than in the experimental model (92-1: 8.8-fold, Mel270: 5.7-fold, UPMD2: 2-fold) (Figure 7). Among all cell lines, however, the spontaneous model displayed a higher selectivity for liver metastasis than for metastasis of the other organs tested, e.g., the eyes, as the eyes developed lower amounts of metastasis in the spontaneous model than in the experimental model (Figure 7). In the spontaneous model, tumors established from 92-1 cells caused the significantly highest amount of liver metastasis, while MM28 cells induced the significantly highest amount of experimental liver metastasis (Figure 7).

Similarly, with all cell lines, the percentage of chick embryos that developed metastases in multiple organs (≥3 organs) was higher in the experimental model (70–92%) than in the spontaneous model (20–67%) (Table 2).

An exception was the UPMD2 cell line, which showed an equally high percentage of chick embryos with multiple organ metastases in both the experimental model (90%) and the spontaneous model (91%) (Table 2). Interestingly, in the spontaneous model, the proportion of chick embryos with solely liver metastases was highest in tumors derived from the Mel270 (70% of chick embryos) and MM28 (40% of chick embryos) cell lines (Table 2).

## 4. Discussion

In our study, we exploited the CAM assay as an in vivo model to quantify the metastatic activity of UM tumor cells in organs such as the liver, lung, and brain. Additionally, we examined orthotropic growth in the eyes and metastasis of the LCAM. We applied two fundamentally different models of induced metastasis, as each model provides different insights into the metastatic cascade. A panel of five different UM cell lines was injected directly into the chick’s circulation to study hematogenous spread and its ability to grow in secondary organs, referred to as an experimental model. In contrast, in the spontaneous model, UM cells were grafted onto the CAM to analyze potential tumor cell escape and the subsequent dissemination of the cells to the secondary organs, which better mimics the natural process of UM metastasis. For the first time, we detected UM tumor cells of primary tumor or hepatic metastases origin by qPCR in various frequencies and amounts in multiple organs of each individual animal. We also included aggressive cell lines that have numerous risk factors for the development of metastases, such as genetic status, particularly the presence of monosomy 3 and BAP1 deficiency.

The experimental approach of our study showed that 92-1 cells migrated most frequently to the LCAM and liver, followed by the eyes, lungs, and brains. Importantly, the liver was predominantly affected, reflecting the early and primary site of metastasis in patients (Figure 1). In addition, during the prolonged incubation period up to ED 18, the amount of metastasis increased during the simultaneous development of the embryonic organs (Figure 1). At the same time, the chick embryo’s immune system begins to develop, which can influence the persistence of metastasis in organs, such as the liver and the lungs during the course of each experiment. Although the immune system of chick embryos is not yet fully functional before ED 18, macrophages can be detected in the liver at ED 12, which are capable of recognizing antigens and triggering chemotaxis [58]. The early development of the liver’s immune system could explain why there was no significant increase in liver metastasis between ED 11 and ED 18. Further, only cell lines 92-1 and Mel270 induced significant amounts of lung metastases (Figure 1 and Figure 2). However, the frequency of 92-1 lung metastasis-positive chick embryos was lower at ED 18 compared to ED 11, suggesting that UM cells did not stably persist in all lungs (Figure 1). One explanation for this finding could be that UM cells, which have not extravasated into the lung tissue to form colonies, have left the lung with the bloodstream and/or have been removed by circulating immune cells by ED 18. Apparently, UM cells have a much stronger ability to remain stable in the livers than in the lungs. Similar to a histological study, no lung metastasis was detectable, while the livers and brains were strongly metastasized by 92-1 or OMM1 cells at ED 14 [59]. However, histologic examination of tissues may not detect diffuse micro-metastases, whereas the qPCR method allows the quantification of submicroscopic metastases in chick embryo organs, including the lungs [72,74,75].

Furthermore, our qPCR analysis revealed that UM cell lines consistently caused the most metastasis in the livers and LCAMs in both experimental and spontaneous CAM models. The stereomicroscopic evaluation revealed highly pigmented metastatic foci in LCAM tissue in 10–50% of the chick embryos in the experimental model and 9–20% of the chick embryos in the spontaneous model, confirming that UM cells survived in the bloodstream and were able to colonize the tissues (Figure 1 and Figure 2). We also observed abnormal green-brown pigmentation of the livers in approximately 10% of metastatic chick embryos at ED 18, indicating progression of metastatic liver disease, which our qPCR analysis underscores (Figure 1 and Figure 2). Additionally, the spontaneous liver metastasis of 92-1 cells was previously confirmed by histologic evidence of single metastatic lesions in the chick embryo liver at ED 14 [76]. However, we did not histologically examine metastatic lesions in the liver because in our study homogenates of each individual whole organ were subjected to qPCR analysis to avoid missing hidden micro-metastasis. In addition, we observed hyperpigmentation of the iris in about 10% of the metastatic chicks, indicating iris melanoma, while no visible pigmentation was observed in the brains or lungs. These results indicate that in the CAM model, the LCAM, liver, and eyes are the organs most affected by UM tumor cells. However, the short incubation period of 11 days may have prevented the formation of macroscopically visible metastases in all of the embryonic organs. Nevertheless, qPCR analysis revealed that the organs of the chick embryo, such as the livers, eyes, brains, and lungs, were colonized to varying degrees by several UM cell lines in either experimental or spontaneous models.

The cell lines 92-1 and Mel270, which originate from primary tumors, theoretically have a low risk of metastasis, as these cells have a genetic disomy 3 status with an EIFAX mutation (92-1) or without an additional secondary driver mutation (Mel270) (Table 1). Despite their genetic status, cell lines 92-1 and Mel270 frequently induced the largest extent of metastasis, resulting in 60% (92-1) and 46% (Mel270) of chick embryos having tumor cells in multiple organs (Figure 1 and Figure 2). The high rate of metastasis in multiple organs could be due in part to the high rate of cell proliferation if the low cell doubling time observed in cell cultures (Table 1) is maintained in the chick embryo organs, albeit to a different extent in each organ due to the unique microenvironment. On the other hand, the cell lines UPMD2, UPMM3, or MM28, which had a significantly higher doubling time than 92-1 or Mel270, developed multiple metastases less frequently, with 40% (UPMD2), 30% (UPMM3), and 20% (MM28) of the chick embryos being metastasis-positive in all organs tested (Figure 2 and Figure 3). Remarkably, despite its relatively low proliferation rate, the MM28 cell line caused the largest amount of liver metastases, which is consistent with its hepatic metastatic origin (Figure 2 and Figure 7). A previous study in a larval zebrafish xenograft model showed that UM cell lines derived from liver metastases, such as OMM2.3 and OMM2.5, exhibit a more aggressive phenotype, leading to increased tumor cell burden and migration compared to cell lines derived from primary tumors, such as Mel270 and 92-1 [77]. Although the metastatic MM28 cell line developed by far the most liver metastases, abnormal brownish tissue lesions were visible in only 10% of cases (Figure 2). This finding could be due to the low pigmentation and/or the more diffuse distribution pattern of MM28 tumor cells in the livers. UM patients may develop liver metastases with a variable number of metastases, leading to either a military or a solitary metastatic pattern detectable on radiology [78]. However, the mutation status of the primary tumor, e.g., the presence of a BAP1 mutation, did not correlate with any of these growth patterns, while the loss of chromosomal 1p and 8p was much more pronounced in patients with military metastasis [78]. In keeping with a potential diffuse growth pattern characterized by innumerable micro-metastases throughout the liver, the MM28 cell line contains a genetic loss of chromosomes 1p and 8p [67]. However, a histological analysis would be essential for investigating the pathology of the metastasized organs. As for the genetic high-risk factors, abnormal chromosome 3 and BAP1 deficiency in MM28, this mutation profile is unlikely to have promoted liver metastasis alone. This assumption is supported by the finding that the primary UPMM3 cell line, which also has monosomy 3 and BAP1 deficiency, did not show a higher rate of metastasis to the liver or metastasis to another organ compared to its disomic and BAP1-positive counterpart UPMD2 (Figure 3 and Figure 7, Table 2). However, the combination of genetic risk factors in the metastatic MM28, such as abnormal chromosomes 8, 3, and BAP1 deficiency, may have contributed to the accelerated induction of liver metastases. The presence of 8q gain was indeed predictive of an even worse prognosis in patients with a BAP1 mutation [79].

In the spontaneous setting, the cell lines formed vascularized tumors with different morphological features on the CAM, which may have influenced the resulting metastasis (Figure 4). Despite some differences in tumor morphology, such as pigmentation, size, vascularization, and stability, the resulting spontaneous metastasis predominantly affected the liver, with all other organs being affected much less (Figure 5 and Figure 6). Accordingly, the chick embryos developed fewer multiple organ metastases, as only 25%, 10%, or 0% of the chick embryos tested positive for all organs with 92-1, Mel270, or MM28, respectively. It is particularly interesting that Mel270 and MM28 cell lines caused solely liver metastases in 70% and 40% of the chick embryos, respectively (Figure 5). Unexpectedly, the number of liver metastases was much lower in MM28 compared to 92-1 or Mel270, regardless of significant differences in primary tumor size or vascularization (Figure 4). However, the larger, flat, and unstable Mel270 tumors may have facilitated the invasion of more tumor cells into the CAM and existing vessels, which may have contributed to the high liver metastasis rate of this cell line compared to MM28. Interestingly, the amount of liver metastasis caused by MM28 was much lower in the spontaneous model than in the experimental model (Figure 7). This finding could be explained by a relatively low invasive or intravasive ability of the MM28 cells, which could have led to a delay in spontaneous liver metastasis. Regarding the genetic status of the cells, a direct comparison of the primary cell lines UPMD2 and UPMM3 revealed that the genetic profile of monosomy 3/BAP1 deficiency did not promote spontaneous metastasis, suggesting that it has no impact on invasive or intravasive ability (Figure 6). In addition, cell lines with a lower proliferation rate, such as MM28, UPMD2, and UPMM3, resulted in a lower amount of liver metastasis than cell lines 92-1 and Mel270. A stronger cellular proliferation of the tumor is also associated with metastasis and a poor prognosis for UM patients [51]. Conversely, low proliferation correlates with prolonged disease-free survival of UM patients with BAP1-positive as well as BAP1-losing tumors [80].

Overall, the spontaneous model showed a higher selectivity for liver metastases compared to the experimental model (Figure 7). However, this effect could be due to the limited incubation period, as the tumor cells need additional time to escape the tumor and enter the bloodstream, which could lead to delayed metastasis in other target organs such as the eyes, brains, and lungs. This conclusion is supported by our initial observation that a prolonged incubation period of chick embryos led to increased metastasis in other organs (Figure 1). In addition, overt signs of organ metastasis were less frequent in the spontaneous model than in the experimental model, suggesting an earlier stage of the metastatic process. An alternative explanation would be that the tumor microenvironment on the CAM generated a more invasive cell type with a higher selectivity for the liver, e.g., by inducing a strong expression of the hepatocyte growth factor receptor c-Met in the tumor cells. High c-Met expression in primary tumors is associated with an increased risk of developing liver metastases in UM patients [81]. The tumor microenvironment on the CAM may also have made the livers more hospitable for metastatic growth prior to colonization, e.g., through the secretion of extracellular vesicles [82]. In our study, however, these liver-selective properties would exist regardless of cytogenetic profile or cell morphology, as tumors of all cell lines caused high rates of metastasis to the liver, albeit to varying degrees (Figure 7). In terms of cell morphology, the epithelioid cell lines 92-1 and UPMD2 showed the highest proportion of chick embryos with spontaneous metastasis in all organs in our study, indicating a higher invasive or intravasive capacity. Moreover, these epithelioid cell lines most frequently targeted the eyes. However, in terms of liver metastasis, epithelioid morphology does not appear to be responsible for the increased amounts, as the Mel270 spindle cell type developed significantly greater amounts of liver metastases than the UPMD2 cell line. Furthermore, similar to clinical cases, the xenografts on the CAM varied in their degree of pigmentation from amelanotic (MM28) to highly pigmented (92-1, UPMD2). Heavy pigmentation of primary tumors is a prognostic risk factor in UM, and several reports have mentioned that heavier pigmentation is often associated with the presence of epithelioid cells [83]. Consistent with these findings in patients, the 92-1, UPMD2, and Mel270 cell lines, which formed pigmented tumors on the CAM, caused greater amounts of liver metastases compared to UPMM3 and MM28 cells, which formed pale or amelanotic tumors on the CAM (Figure 4).

In patients, the cytogenetic profiles with chromosome 3 aberration, BAP1 loss, and chromosome 8q gain are strong prognostic risk factors for the development of liver metastases [35,84]. However, the biological causality between cytogenetic status and metastasis remains unclear, and few studies have addressed the contribution of these genetic risk factors to primary tumor growth and metastasis in animal models [73,85]. Unexpectedly, experiments with BAP1-depleted cell lines 92-1 and OCM1A injected into NOD-SCID-gamma mice showed less primary tumor growth in the flanks and less metastasis to the liver and lungs after injection into the tail veins. The study indicates that BAP1 deficiency does not cause increased proliferation, invasion, or tumorigenicity [86]. In another study, knockout of BAP1 in GNA11 mutant mice did not lead to an increase in the size or frequency of uveal pathology and lung lesions, and no pigmented liver lesions were observed [87]. A recent study addressed the combinatorial effects of secondary driver mutation SF3B1 and BAP1. The simultaneous occurrence of SF3B1 mutations and BAP1 deficiency in Mel202 cells reduced their invasion capacity in the zebrafish xenograft model. However, BAP1 deficiency in metastatic OMM2.3 (SF3B1 wild type) did not alter invasion compared to OMM2.3 control, indicating that BAP1 deficiency alone had no effect on invasion ability [88]. Also, in our spontaneous CAM xenograft model, transplantation of BAP1-deficient UPMM3 or MM28 cells did not result in significantly larger tumors or increased spontaneous metastasis to the liver and lungs compared to BAP1-positive cells. However, cell types containing the genetic risk factor chromosome 8q gain resulted in the highest amounts of liver metastases in either the experimental model (MM28) or the spontaneous model (92-1), suggesting that 8q gain may be associated with a high metastatic potential in the model. A recent study of advanced UM confirmed the frequent presence of 8q gain (85%), monosomy 3 (46%), and altered BAP1 (62%) in resected metastases [89]. However, similar to other animal models, monosomy 3 and BAP1 deficiency could not yet be confirmed as a major risk factor for increased tumor growth and metastasis in our CAM model, suggesting the involvement of additional factors or conditions in patients that are not present in in vivo models. In contrast to our study, primary tumors in patients often contain a mixture of tumor cells with different genetic statuses, morphology, pigmentation, or proliferation rates as well as immune cells [90]. The complexity of cell-cell interactions in the tumor microenvironment may trigger the invasion and migration of tumor cells with monosomy 3 and BAP1 deficiency. Further studies that include larger groups of cell lines with different genetic profiles and/or that address the heterogeneity of primary tumors by using cell mixtures or patient-derived tumor grafts in the animal models could overcome the limitations of the study.

## 5. Conclusions

Our study suggests that the CAM xenograft model is suitable for investigating both experimental and spontaneous UM metastasis. We were able to show for the first time that transplanted UM cells spread to multiple organs, with liver metastasis predominating and occurring early. Evaluation of metastasis frequency and quantity by qPCR analysis revealed a cell-specific spectrum of organ metastasis. UM cells derived from primary tumors or liver metastases contained multiple risk factors potentially associated with metastasis. Our study suggests that a high proliferation rate, hyperpigmentation, and an epithelioid cell type are associated with severe metastasis involving multiple organs such as the eyes, liver, brain, lungs, and the LCAM in the presented UM model. In addition, the origin of liver metastases and the abnormal genetic status of chromosome 8 rather than aberrant chromosome 3 and BAP1 deficiency appeared to be related to the severity of liver metastases. The CAM xenograft model may prove useful to study the metastatic potential of patients or to test individualized therapeutic options for metastasis in different organs.

## Figures and Tables

**Table 1:** Characteristic of uveal melanoma cell lines.

Cell Line	Origin	Primary/Secondary Driver Mutations	Status Chromosome 3/8	Morphology	Doubling Time	References
92-1	Primary untreated	GNAQ Q209L/EIF1AX	Disomy 3/Gain 8q	Epithelioid	38–58 h	[63,64]
Mel270	Primary recurrence	GNAQ Q209P/WT	Disomy 3, Loss 3p24, 3q21.2-3q22/Disomy 8q, extra 8	Spindle	43 h	[64,65,66]
MM28	Hepatic metastasis	GNA11 Q209L/BAP1 *	Loss 3q/Gain 8q, Loss 8p	Mixed	109 h	[67]
UPMD2	Primary untreated	GNA11 Q209L/WT.	Disomy 3/n.d.	Epithelioid	150 h	[68,69,70]
UPMM3	Primary untreated	GNAQ Q209P/BAP1 *	Monosomy 3/n.d.	Mixed	100–150 h	[68,69,70]

* BAP1 protein loss, n.d. not determined.

**Table 2:** Percentages of chick embryos with multiple organ metastasis in experimental or spontaneous model.

Cell Type	Experimental ≤2 Organs	Experimental ≥3 Organs	Spontaneous ≤2 Organs	Spontaneous ≥3 Organs
92-1	20%	80%	33% (15% eyes only)	67%
Mel270	8%	92%	80% (70% liver only)	20%
MM28	20%	80%	60% (40% liver only)	40%
UPMD2	10%	90%	9%	91%
UPMM3	30%	70%	40% (10% liver only)	60%

## Data Availability

The data presented in this study are contained within the article.
